# Hypoxic Physiological Environments in a Gas-Regulated Microfluidic Device

**DOI:** 10.3390/mi10010016

**Published:** 2018-12-28

**Authors:** Insu Lee, Jin Hyuk Woo, Min Lee, Tae-Joon Jeon, Sun Min Kim

**Affiliations:** 1 Department of Mechanical Engineering, Inha University, Incheon 22212, Korea; islee0929@gmail.com (I.L.); dnwlsgur1213@gmail.com (J.H.W.); 2 Division of Advanced Prosthodontics, University of California at Los Angeles, 10833 Le Conte Avenue, Los Angeles, CA 90095, USA; leemin@ucla.edu; 3 Department of Bioengineering, University of California at Los Angeles, 420 Westwood Plaza, Los Angeles, CA 90095, USA; 4 Department of Bioengineering, Inha University, Incheon 22212, Korea

**Keywords:** hypoxic condition, microfluidic system, computational simulation, oxygen detection, oxygen scavenger

## Abstract

Hypoxic environment is known as one of the critical factors in various physiological/pathological processes. It is imperative to recapitulate oxygen level in microscale for human physiology/pathology induced by hypoxia. Herein, we propose an oxygen-regulating system that can be applied to in vitro tissue models. We fabricated a microdevice with a gas-permeable membrane, allowing oxygen diffusion without direct contact to cells. We verified the formation of oxygen level less than 2% O_2_ concentration inside the device through computational simulation and experiments. H9c2 heart myoblasts were exposed to hypoxic condition in the device, and their cell viability were investigated. We anticipate that our system will be integrated with a platform to study hypoxia-induced human physiology and pathology as an efficient oxygen-regulating system.

## 1. Introduction

Human tissues and organs are exposed to hypoxic environment due to its critical role in various physiological and pathological processes. For example, erythropoiesis [1] is triggered by hypoxic condition, and hypoxia causes brain stroke [2] and cardiac infarction [3], which induces severe tissue damages. Hypoxia has been previously investigated on animal [4,5] or static dish culture models [6] for biological studies. These models typically utilized bulky gas regulators and reservoirs to establish hypoxic condition, or oxygen scavenger was mixed with culture medium and directly applied to the cells. However, these methods have several limitations in recapitulating the in vivo microenvironments of tissues and organs. The bulky gas-regulating instruments complicate system setup, operation, and industrial applications. Moreover, the direct contact of oxygen scavenger to cells adversely affects cell viability due to its cytotoxicity. Lab-on-chips with different types of gas-permeable membrane [7,8], multilayered chip structure [9], or complex microchannel-integrated devices [10] have been proposed to overcome these hurdles [11]. However, complicated geometry and additional gas-regulating instruments were still a burden. In this research, we propose a microfluidic system that can induce hypoxic condition to cells and tissues in microscale. Using oxygen sensor film and integrated oxygen-scavenging channel in the device, we were able to indirectly monitor oxygen level in situ and control oxygen concentration in the desired ranges. The microfluidic system comprises multilayered microchannels separated by a polydimethylsiloxane (PDMS) membrane for cell culture and oxygen scavenging. The PDMS membrane permits oxygen diffusion [12] without the direct contact between oxygen scavenger and cells, preventing the cytotoxicity issue. First, we performed numerical calculations to verify oxygen regulation in our microfluidic system using multiphysics software. Then, hypoxia formation in the device was validated through oxygen sensor. We validated the applicability of our device for recapitulating hypoxia-related pathologies by culturing H9c2 heart myoblasts inside the device. We induced hypoxic condition to the cells on a specific region and compared the cell viability between the normoxia and hypoxia regions. 

## 2. Materials and Methods 

### 2.1. Device Fabrication

**Figure 1:**
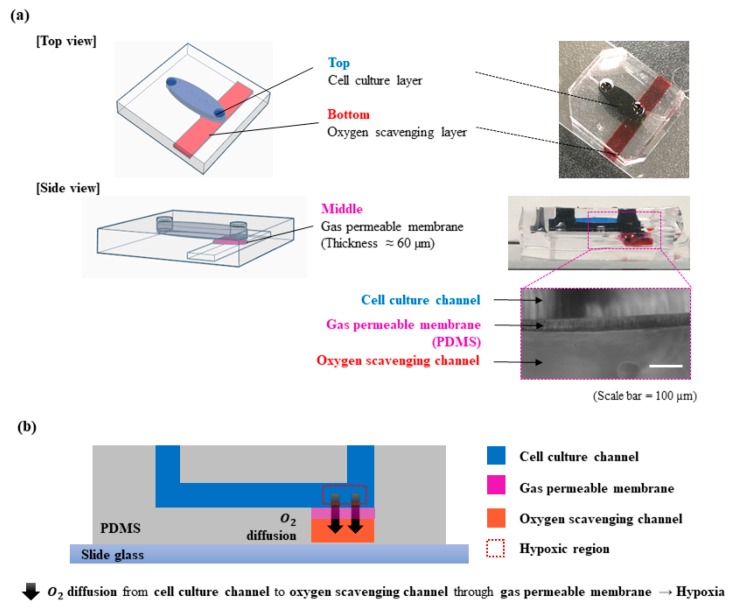
Schematic images of the designed microfluidic device capable of controlling oxygen level. (**a**) Geometry of the microfluidic device; (**b**) oxygen-scavenging process in the microfluidic device.

**Figure 2:**
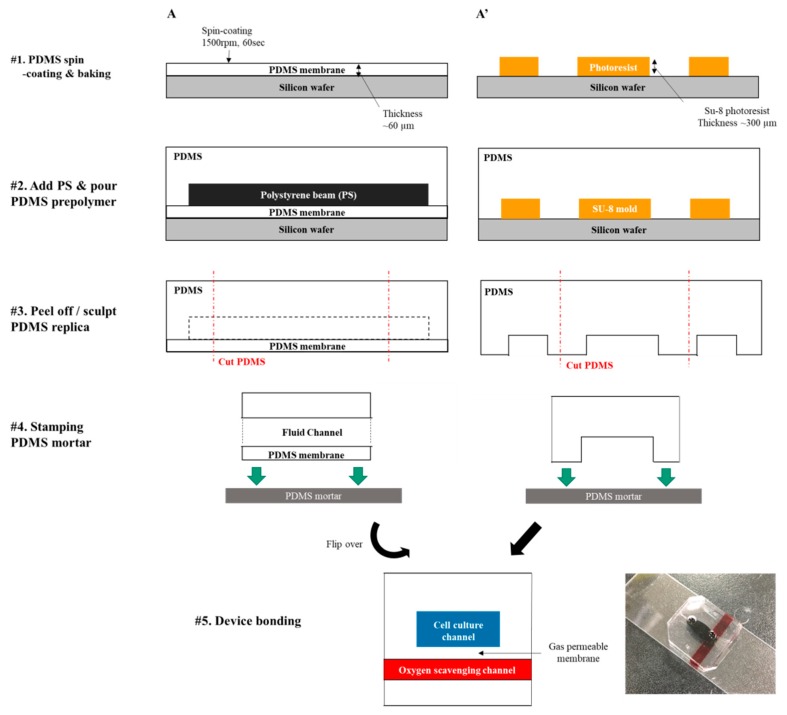
Fabrication steps of the microfluidic device using soft lithography.

The device with three layers was designed using CAD software (AutoCAD, Autodesk, San Rafael, CA, USA). The top layer is a cell culture channel for culturing cells and extracellular matrix, the middle layer is a PDMS membrane for gas exchange, and the bottom layer is an oxygen-scavenging layer to absorb oxygen (Figure 1). The device was fabricated using general soft lithography process (Figure 2). We used SU-8 photoresist (SU-8 2100, MicroChem, Westborough, MA, USA) and achieved 0.3-mm-thick mold to fabricate the cell culture channel of the device. On the SU-8 mold, we fabricated two layers of PDMS (Sylgard 184, Dow Corning, Midland, MI, USA). One layer contains only cell culture channel, while the other layer includes PDMS membrane and oxygen-scavenging channel. PDMS mixture (elastomer: curing agent = 10:1 weight ratio) was poured on the bare SU-8 mold to fabricate the cell culture channel layer. Then, the PDMS mixture was polymerized through baking process at 65 °C in a convection oven (NDO-400, Eyera, Tokyo, Japan). For fabrication of the layer with a PDMS gas-permeable membrane and oxygen-scavenging channel, PDMS mixture (elastomer: curing agent = 10:1 weight ratio) was spin-coated (1000 rpm for 90 s) on a 4-inch silicon wafer to form a layer of 50–60 µm thickness [13]. After the baking process, polystyrene (PS) beam (25 mm × 4 mm × 1 mm = length × width × height) was added on top of the polymerized PDMS membrane layer, and extra PDMS mixture was poured on to the wafer. The PS beam was pulled out after the baking process to form the oxygen-scavenging channel. Both layers were attached by PDMS mortar on the side that had the cell culture channel layer [14]. PDMS mortar was fabricated with spin-coated (3000 rpm for 300 s) PDMS mixture (elastomer: curing agent = 10:3 weight ratio). We used a biopsy punch (Miltex®, Ted Pella, Redding, CA, USA) to make inlet/outlet ports for fluid transport.

### 2.2. Numerical Calculation of Oxygen Concentration in the Device 

We used multiphysics simulation software (COMSOL Multiphysics, COMSOL, Los Angeles, CA, USA) to evaluate the oxygen control performance using oxygen scavenger inside the device. We applied the same CAD model used for device fabrication. 2D incompressible flow condition of the cell culture channel was set as 1 μL/min of air-saturated water from inlet to outlet. The boundary conditions for the flow channel surfaces were set as a no-slip condition. We considered cell culture medium as water, which has 1 g/cm^3^ of density and 1 cP of viscosity. The top oxygen-scavenging channel was set as 0% O_2_ due to the rapid reaction of oxygen scavenger with catalyst (cobalt nitrate) [15]. Velocity profile and oxygen concentration in the cell culture channel were calculated.

### 2.3. Oxygen Level Control in the Microfluidic Device

We used an optical oxygen sensor film (Oxygen Sensor Foil SF-RPSu4, PreSens Precision Sensing GmbH, Regensburg, Germany) and an optical oxygen detector (VisiSens™, PreSens Precision Sensing GmbH). The sensor film was attached on top of the PDMS membrane using PDMS mortar. Device operation was conducted in a mini-incubator (LICES, N-biotek, Bucheon-si, Korea), which maintained the temperature and humidity at 37 °C and 50%–60%, respectively. Through this process, we measured the oxygen concentration of the cell culture layer. The oxygen scavenger was composed of 1 g of sodium sulfite (Na_2_SO_3_) (S0505, Sigma Aldrich, Saint Louis, MO, USA) and 50 µL of cobalt nitrate (Co(NO_3_)_2_) standard solution (170313, Milipore, Burlington, MA, USA). Oxygen measurement was carried out for 12 h in 10 min intervals. Air-saturated water was pumped into the cell culture channel at a rate of 1 µL/min, and the oxygen scavenger was pumped into the oxygen-scavenging channel at a rate of 30 µL/min using a syringe pump (LEGATO® 270, KDscientific, Holliston, MA, USA).

### 2.4. Oxygen Level Measurement

The time lapse images were captured to calculate time-dependent oxygen concentration through image processing/analysis software. For data calibration, we used 2-point calibration with oxygen scavenger as the low point and air-saturated water as the high point. Air-saturated water was achieved through aeration of DI water using piston pump (2522C-10, Welch, Concord, MA, USA) at room temperature. With air-saturated water, the oxygen sensor film presented green fluorescence due to oxygen quenching; with oxygen-free water, the oxygen sensor film presented red fluorescence. Less than one minute after injecting the oxygen scavenger, the color of the oxygen sensor film changed. After calibration, we measured the oxygen level on top of the membrane region for 12 h in 5- or 10-min intervals. The measurement was conducted in light-protected condition.

### 2.5. Cell Culture 

H9c2 rat heart myoblast (21446, SNU cell bank, Seoul, Korea) were cultured in a 75-mL cell culture flask (Corning, Corning, NY, USA) with 10% fetal bovine serum (FBS) (10082147, Gibco, Waltham, MA, USA) and 1% penicillin/streptomycin (15140122, Invitrogen, Carlsbad, CA, USA) in Dulbecco’s modified Eagle’s medium (DMEM) (11965092, Invitrogen). Then, the cell culture flask was incubated in a 5% CO_2_ at 37 °C incubator (HeraCell 150, Thermo Fisher Scientific, Waltham, MA, USA) for 2–3 days until the cell population reached less than 80%–90% of the flask. Cells were harvested upon trypsinization using 0.05% trypsin–EDTA (25200-056, Gibco). 

### 2.6. Cell Culture under Hypoxic Condition

Harvested H9c2 myoblasts were seeded into the cell culture channel of the microfluidic device (cell density = 4M cells/mL). Cell-seeded microfluidic devices were cultured for 1 day in the incubator. Oxygen scavenger was applied to the cell culture layer to form hypoxic condition for 6 h. The myoblasts were stained after being exposed to hypoxic condition using LIVE/DEAD™ Viability/Cytotoxicity Kit (L3224, Thermo Fisher Scientific) [16] to confirm their viability in different oxygen conditions.

## 3. Results and Discussion

### 3.1. Numerical Calculation of Oxygen Concentration Profile in the Microfluidic Channel

**Figure 3:**
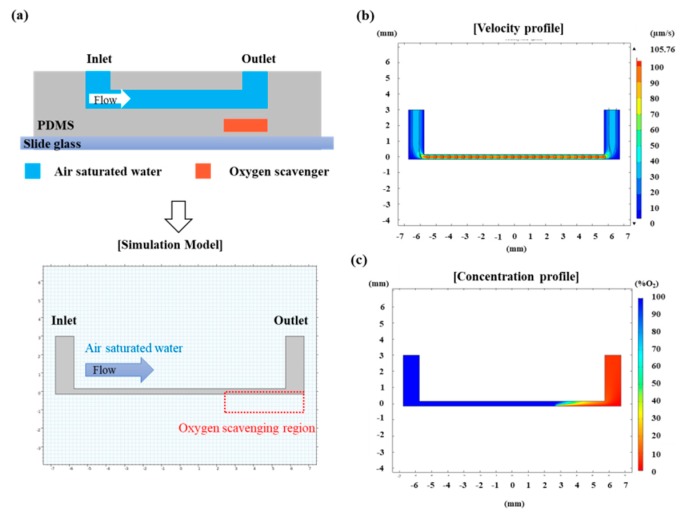
Numerical calculations of the oxygen concentration in the microfluidic device. (**a**) Modeling of 2D flow in the cell culture channel under oxygen diffusion; (**b**) flow velocity profile; and (**c**) oxygen concentration profile in the cell culture channel.

We numerically calculated the oxygen concentration in the designed device using COMSOL software. The structure and size of the model was identical to the CAD design of the device (Figure 3a). The concentration profile was calculated by solving 2D convection and diffusion equation [9].

$$\frac{\partial c}{\partial t}=\nabla \cdot \left(D\nabla \cdot c\right)-\nabla \cdot \left(\vec{V}c\right)$$

We assumed the steady state, and the diffusion coefficient of water was set as 1.9 × 10^−9^ m^2^∙s^−1^. We did not consider oxygen transport through the PDMS wall of microchannels because the thickness of PDMS walls is enough to prevent oxygen transport. The environment of the cell culture channel was set as air-saturated water flow condition to mimic cell culture media supply condition. Oxygen concentration of the PDMS membrane region was assumed as 0% O_2_, considering the rapid catalytic reaction of the oxygen-scavenging process. We applied 1 µL/min flow-rate in the cell culture channel that was designed for cell culture medium supply. Velocity profile (Figure 3b) and oxygen concentration (Figure 3c) inside the cell culture channel was investigated. The velocity profile in the microfluidic channel presented a fully developed laminar flow. For oxygen concentration, the end part of the cell culture channel was calculated as less than 10% air-saturated, which is below 2% O_2_ concentration due to oxygen diffusion. The result confirmed that we were able to control the O_2_ concentration below 2% O_2_, which corresponds to the hypoxic condition in tissues [17].

### 3.2. Recapitulation of Hypoxic Condition in the Device

**Figure 4:**
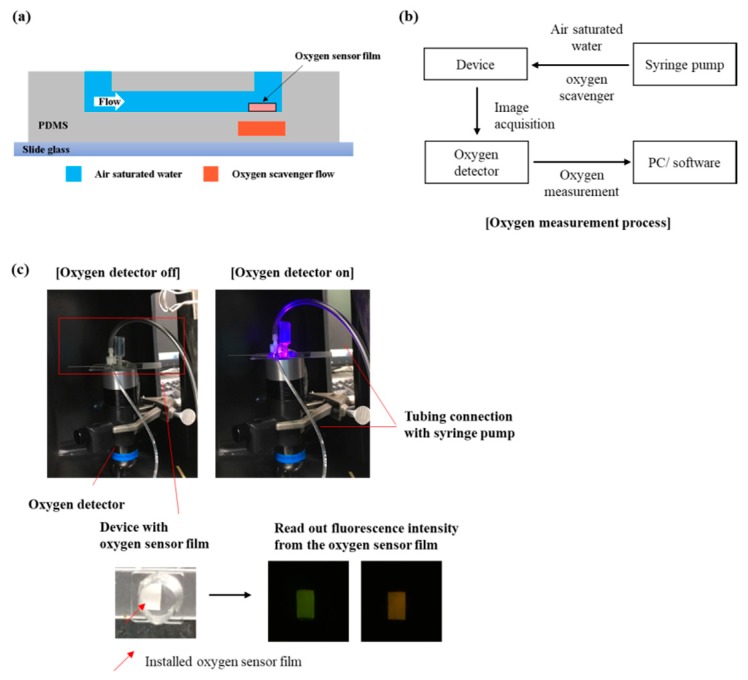
Oxygen level monitoring in the microfluidic device. (**a**) Schematic image of oxygen sensor film integrated device; (**b**) experimental setup for oxygen monitoring in the microfluidic device; (**c**) Experimental setup for measuring oxygen level with oxygen sensor film integrated microfluidic device.

We generated the hypoxic condition inside the microfluidic device under normal cell culture environment. We attached oxygen sensor film to the preassigned channel surface and carried out the experiment to measure the oxygen level in the device (Figure 4a). Experimental setup consisted of four main parts: device, syringe pump, oxygen detector, and PC/software (Figure 4b). 

**Figure 5:**
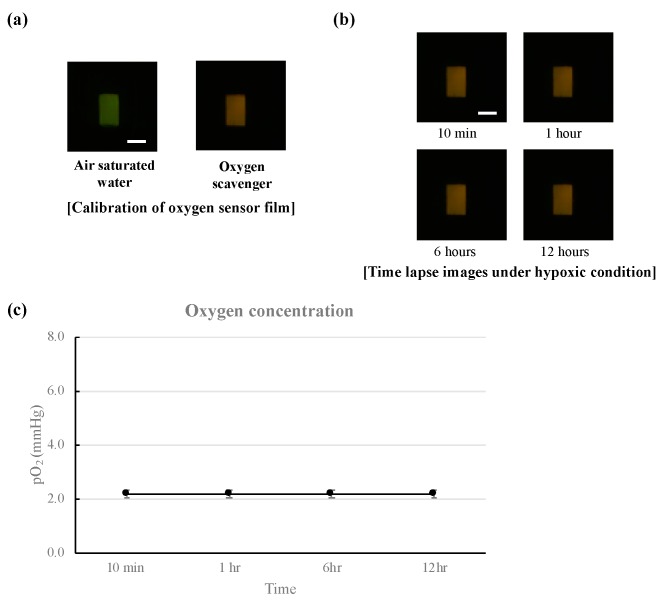
Validation of long-term hypoxia formation in the microfluidic device using oxygen sensor film and detector. (**a**) Calibration for oxygen detector; (**b**) time-lapse images of oxygen sensor film under hypoxic condition; (**c**) oxygen concentration measured with the time-lapse images.

The device was operated inside a mini-incubator. We captured the time-lapse fluorescent images of the oxygen sensor film in 10-min intervals for 12 h with the oxygen detector. We could visibly monitor low-level oxygen concentration in the device with red fluorescent image (Figure 5b). Figure 5c shows the oxygen level measured by time-based fluorescence intensity values from the oxygen sensor film. The measured oxygen level showed 2.2 mmHg pO_2_ on average (standard deviation = 0.1), which confirmed the formation of hypoxic condition in the cell culture channel. These oxygen level can be considered as in vivo tissue hypoxia [17].

### 3.3. Cell Viability Assay under Hypoxic Condition 

**Figure 6:**
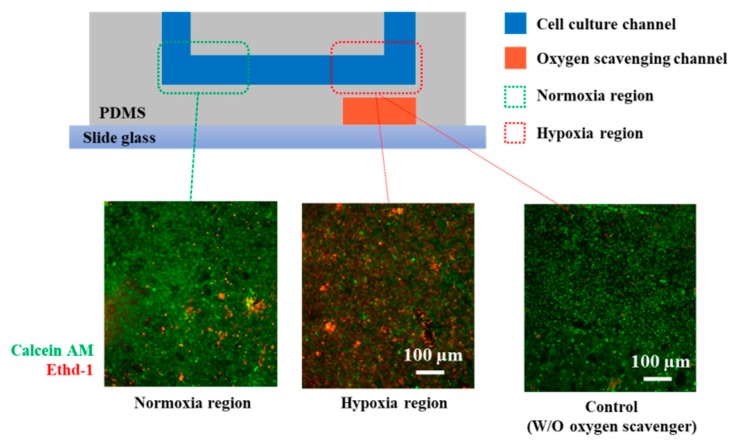
Live/dead assay of H9c2 heart myoblasts under normoxia and hypoxia conditions. H9c2 heart myoblasts were cultured on the normoxia and hypoxia regions in the microchannel. After 6 h of hypoxic condition, cells were stained for live/dead assay. Calcein AM (green fluorescence) presents live cells, and ethidium homodimer-1 (red fluorescence) presents dead cells.

H9c2 heart myoblasts were cultured in the microfluidic device under hypoxic condition. Cell viability was investigated using the live/dead staining method to confirm hypoxic effects on H9c2 heart myoblast (Figure 6). Six hours of hypoxic condition was provided to the cells to mimic ischemic environment of the heart [18,19,20,21]. We monitored two different regions of the cell culture channel: normoxia and hypoxia regions. Live and dead cells were stained with calcein AM (green color) and ethidium homodimer-1 (red color), respectively. The hypoxia region (on top of the oxygen-scavenging layer) remained with lower cell viability than the normoxia region (far from the oxygen-scavenging layer). 

## 4. Conclusions

In this research, we developed a microfluidic system that can control the oxygen level in microscale without cell toxicity issues for hypoxia study. Flow condition and oxygen concentration profile in the microchannel were investigated and validated by numerical calculations. Oxygen regulation in the device was experimentally confirmed by oxygen-detecting instruments. Heart myoblasts were cultured in the device with hypoxic condition to validate its performance as a study model. We believe that our system can be employed as an efficient research platform for studying physiology/pathology related with oxygen concentration.
